# Increased local concentration of complement C5a contributes to incisional pain in mice

**DOI:** 10.1186/1742-2094-8-80

**Published:** 2011-07-07

**Authors:** Jun H Jang, Deyong Liang, Kanta Kido, Yuan Sun, David J Clark, Timothy J Brennan

**Affiliations:** 1 Department of Anesthesia, University of Iowa Hospitals and Clinics, Iowa City, Iowa, USA; 2 Department of Anesthesia, Veterans Affairs Palo Alto Healthcare System and Stanford University School of Medicine, Stanford, California, USA; 3 Department of Pharmacology, University of Iowa Hospitals and Clinics, Iowa City, Iowa, USA

## Abstract

**Background:**

In our previous study, we demonstrated that local injection of complement C5a and C3a produce mechanical and heat hyperalgesia, and that C5a and C3a activate and sensitize cutaneous nociceptors in normal skin, suggesting a contribution of complement fragments to acute pain. Other studies also have shown that the complement system can be activated by surgical incision, and the systemic blockade of C5a receptor (C5aR) reduces incision-induced pain and inflammation. In this study, we further examined the possible contribution of wound area C5a to incisional pain.

**Methods:**

Using of a hind paw incisional model, the effects of a selective C5aR antagonist, PMX53, on nociceptive behaviors were measured after incision *in vivo*. mRNA levels of C5 and C5aR in skin, dorsal root ganglia (DRG) and spinal cord, and C5a protein levels in the skin were quantified after incision. The responses of nociceptors to C5a were also evaluated using the *in vitro *skin-nerve preparation.

**Results:**

Local administration of PMX53 suppressed heat hyperalgesia and mechanical allodynia induced by C5a injection or after hind paw incision *in vivo*. mRNA levels of C5 and C5aR in the skin, but not DRG and spinal cord, were dramatically increased after incision. C5a protein in the skin was also increased after incision. *In vitro *C5a did not increase the prevalence of fibers with ongoing activity in afferents from incised versus control, unincised skin. C5a sensitized C-fiber afferent responses to heat; however, this was less evident in afferents adjacent to the incision. PMX53 blocked sensitization of C-fiber afferents to heat by C5a but did not by itself influence ongoing activity or heat sensitivity in afferents innervating control or incised skin. The magnitude of mechanical responses was also not affected by C5a in any nociceptive fibers innervating incised or unincised skin.

**Conclusions:**

This study demonstrates that high locally generated C5a levels are present in wounds for at least 72 hours after incision. In skin, C5a contributes to hypersensitivity after incision, but increased responsiveness of cutaneous nociceptors to C5a was not evident in incised skin. Thus, high local concentrations of C5a produced in wounds likely contribute to postoperative pain.

## Background

The complement system is a biochemical cascade within the immune system most commonly associated with the enhancement of inflammation and direct attack of foreign organisms. Upon activation of the complement system, split fragments C5a and C3a augment inflammatory responses, e.g. increase blood flow and vascular permeability and facilitate migration of neutrophils and monocytes to the inflamed tissues. C5a and C3a also induce mast cells to release histamine and tumor necrosis factor-α (TNF-α), which contribute to the proliferation of the inflammatory response [1-4]. Other components of the local inflammatory response have been recognized to play roles in pain including cytokines, neuropeptides and neurotrophins [5]. Complement fragments such as C5a may share this property.

Our previous study showed that local injection of complement C5a and C3a produce mechanical and heat hyperalgesia *in vivo*, and that C5a and C3a activate and sensitize cutaneous nociceptors in normal skin *in vitro *[6], suggesting complement fragments may contribute to pain. Furthermore, it has been shown that the complement system can be activated by surgical incision [7-9], and the systemic blockade of C5a receptor (C5aR) reduces incisional allodynia, edema and cytokine expression [10] implying a significant contribution of C5a to the inflammation and pain caused by incision.

Additional studies demonstrate that nociceptors immediately adjacent to the incision sequester NGF, have increased heat sensitivity and increase acid responsiveness [11-14]. However, important questions like whether sensitivity to complement fragments is altered in incised tissue or whether local populations of C5aR support incisional pain behaviors have remained unanswered. In this study, we tested whether the selective C5aR antagonist PMX53 can reduce incisional nociception when injected into peri-incisional skin *in vivo*, and if the responses of nociceptors to C5a are enhanced in incised skin when applied on the peripheral nociceptor terminals *in vitro*. We also examined whether the mRNA levels of C5 and C5aR are altered in skin, dorsal root ganglia (DRG) and spinal cord by incision in an attempt to further localize the likely site of action of C5a in supporting nociception after incision. C5a production in the skin after incision was directly measured as well.

## Materials and methods

### Animals

Male C57BL/6J mice (20-30 g and 6-12 weeks of age, Jackson Labs) that were housed in groups of 4-5 were used. Food and water available *ad libitum *under a 12-h light/dark cycle. Experimental protocols were approved by The Animal Care and Use Committees of the University of Iowa and the VA Palo Alto Health Care System.

### Drug preparation and administration

Recombinant mouse C5a was purchased from R&D Systems, Inc. (Minneapolis, MN). The selective C5aR antagonist PMX53 (AcF-[OPdChaWR]) was the kind gift of Promics (Queensland, Australia). All drugs were dissolved in sterile 0.9% normal saline prior to use [10], and the PBS-vehicle was prepared in the same manner without adding drug.

For local injection of C5aR antagonist in the incisional model, injection (30 μg/15 μl) was performed 1 hour prior to paw incision. The same administration protocol was repeated 2 hours before each test session in the time course studies. The 2 hour time point was the point of maximal analgesic effect as determined in preliminary studies. For studies involving antagonism of the direct local effects of C5a, intraplantar injection of 10 μl C5a solution (200 ng) was accomplished using a 30 gauge needle and a microsyringe (Hamilton, Reno, NV). In these studies involving the systemic administration of C5aR antagonist, the concentration of PMX53 was adjusted so that a 3 mg/kg dose could be administered subcutaneously in the skin of the back in a 100 μl volume; PMX53 was administered 30 minutes prior to local C5a administration. This dose of PMX53 had been shown to be effective and selective in other studies [10,15]. Vehicle injections were used in all studies.

In the electrophysiology study, we tested C5a 0.1 and 1 μg/ml. Because only one concentration of C5a could be applied to each fiber, full dose-response curves were not attempted. The higher concentration sensitized C-fibers to heat in normal skin in our previous study [6]. The lower C5a concentration was included in this study to determine if there were enhanced responses in incised tissue. To ensure C5aR antagonism in the *in vitro *preparation, 0.9 μg/ml (1 μM) PMX53 was selected based on previous observations evaluating the potency of this compound in blocking C5a-induced leukocyte activation [16].

### Surgical incision

Mice were anesthetized with 1.5-2.0% isoflurane delivered through a nose cone, and the hindpaw of mouse was sterilized with 10% povidone-iodine. Plantar incision of hind paw in mice was performed as described previously by our labs [10,17-19]. Briefly, a 0.5 cm incision was made through skin and fascia of the plantar hindpaw with a number 11 scalpel blade. The incision was started 0.2 cm from the proximal edge of the heel and extended distally. The underlying muscle was elevated with curved forceps, leaving the muscle origin and insertion intact. After hemostasis, the wound edges were opposed with a single 6.0 nylon mattress suture and the wound was covered with antibiotic ointment. For hairy skin incision, 0.5 cm incision was made with a number 11 surgical blade on the dorso-medial skin of the left hindpaw in the area innervated by the saphenous nerve. The skin was then closed with two subcutaneous mattress sutures with 6-0 nylon and covered with an antibiotic ointment. In some cases, the skin from both the left and right hindpaws were incised and utilized to minimize the number of animals required for the fiber recordings. In both plantar and hairy skin incision, control mice without incision underwent a sham procedure that consisted of anesthesia, antiseptic preparation, and application of the antibiotic ointment without an incision.

### Behavioral studies

#### Mechanical allodynia

Mechanical nociceptive thresholds were assayed using von Frey filaments according to the "up-down" algorithm described by Chaplan et al. [20] as we described previously [21,22]. Mice were placed on wire mesh platforms in clear cylindrical plastic enclosures of 10 cm diameter and 30 cm height. After 20 minutes of acclimation, von Frey fibers of sequentially increasing or decreasing stiffness were applied to the plantar surface of the right hindpaw and left in place for 5s using sufficient force to slightly bend the filament. Withdrawal of the hindpaw from the fiber was scored as a response. When no response was obtained, the next higher force in the series was applied to the same paw. If a response was obtained, a less stiff fiber was next applied. Testing proceeded in this manner until 4 fibers had been applied after the first one causing a withdrawal response allowing the estimation of the mechanical withdrawal threshold using a curve fitting algorithm [23].

#### Heat hyperalgesia

Paw withdrawal response latencies to noxious heat stimulation were measured using the method of Hargreaves et al. [24] as we have modified for use with mice [21]. In this assay, mice were placed on a temperature-controlled glass surface (29°C) in a clear plastic enclosure similar to those described for mechanical testing. After 30 min of acclimation, a beam of focused light was directed towards the same area of the hindpaw as described for the von Frey assay. A 20 s cutoff was used to prevent tissue damage. In these experiments, the light beam intensity was adjusted to provide an approximate 10 s baseline latency in control mice. Three measurements were made per animal per test session separated by at least one minute.

### Analysis of C5 and C5aR mRNA

Mice were sacrificed at specific time points by CO_2 _asphyxiation. The skin tissue surrounding the incision with approximate 1.5-mm margins was excised. Spinal cord lumbar segments (L3-S1) were harvested by extrusion and rapid dissection on a pre-chilled surface. Dorsal root ganglia (DRG) were dissected using low power binocular magnification. All tissues were quick frozen in liquid nitrogen and stored at -80°C.

For RNA and real-time polymerase chain reaction (PCR), total RNA was isolated from skin, spinal cord and DRG using the RNeasy Mini Kit (Qiagen, Valencia, CA) according to the manufacturer's instructions. The purity and concentration were determined spectrophotometrically. The mRNA samples were reverse transcribed into complementary DNA (cDNA) using a First Strand cDNA Synthesis Kit (Invitrogen, Carlsbad, CA). Real-time PCR was performed in an ABI prism 7900HT system (Applied Biosystems, Foster City, CA). All PCR experiments were performed using the SYBR Green I master kit (Applied Biosystems). The primer sets for C5aR and 18S mRNA and the amplification parameters were described previously [10]. Melting curves were performed to document single product formation and agarose electrophoresis confirmed product size. The C5a primers were purchased from SABiosciences (SABiosciences, Valencia, CA). As negative controls, RNA samples that were not reversibly transcribed were run. Data were normalized by 18S mRNA expression.

### Analysis of C5a in the skin

#### Skin tissue harvest and protein isolation

Mice were euthanized at the time points specified in the figures. The skin tissue surrounding incision with approximate 1.5 mm margins was excised, and skin specimens were placed into phosphate-buffered saline containing a protease inhibitor cocktail (Roche Complete, Roche Diagnostics, Mannheim, Germany), and frozen at -80°C until analysis. For protein isolation these samples were first cut into small pieces with microscissors then disrupted using a Polytron Device (Brinkmann Instruments Inc, Westbury, NY). The samples were then centrifuged at 10,000 rpm at 4°C for 10 min. The supernatant was carefully pipetted into a fresh 1.5 ml tube, which was the material used for protein analysis. Protein concentration was evaluated with a DC Protein Assay kit (Bio-Rad Laboratories, Hercules, CA).

#### C5a assessment

The complement anaphylatoxin C5a level in the skin of the wound site was determined by a mouse C5a DuoSet ELISA Kit according to the instruction provided by the company (R&D Systems, Minneapolis, MN). Briefly, the ELISA plate was coated with capture antibody, and followed by washing with washing buffer. After blocking of non-specific binding sites with blocking buffer, the plate was incubated with 100 μl of each sample or C5a standard in duplicate. After incubation and subsequent washing, detection antibody was added, followed by washing and incubation with streptavidin-HRP at 400 ng/ml. Substrate Solution was added into each well for the color reaction, and the reaction was stopped by addition of stop solution and the absorbance read at 450 nm in microplate reader.

### *In vitro *single fiber recordings

#### Preparation

One day after hairy skin incision or sham incision, mice were killed using CO_2 _inhalation, and the hairy skin from the dorso-medial hind paw was shaved and excised with the innervating saphenous nerve. Attached connective tissue, muscle and tendon were removed. To ensure a sufficient length of axon for recording, the nerve was dissected up to the lumbar plexus. The dissected skin-nerve preparation was fixed in a perfusion chamber with the epidermal side down. The chamber was continuously superfused with a "synthetic interstitial fluid" (SIF) [25], a solution consisting (mM) of 107 NaCI, 26.2 NaHCO_3_, 9.64 sodium gluconate, 5.5 glucose, 7.6 sucrose, 3.48 KCI, I .67 NaH_2_PO_4_, 1.53 CaCl_2_, 0.69 MgSO_4 _at 31 ± 1°C, which was bubbled with a mixture of 95% O_2 _and 5% CO_2_. The nerve, attached to the skin, was drawn through one small hole to the recording chamber, which was filled with paraffin oil. The nerve was placed on a fixed mirror, the sheath was removed and nerve filaments repeatedly teased to allow single fiber recordings to be made using platinum electrodes, one for recording and the other for reference. The reference electrode was grounded to the perfusion chamber. Single nociceptive afferent fibers were recorded extracellularly with a differential amplifier (DAM50, Harvard Apparatus, Holliston, MA). Neural activity was amplified and filtered using standard techniques. Amplified signals were relayed to an oscilloscope and an audio monitor and then into PC computer via a data acquisition system (spike2/CED1401 program). Action potentials collected on a computer were analyzed off-line with a template matching function of spike 2 software (version 4.24).

#### Fiber classification

The search strategy was mechanical stimulation by a fire-polished glass rod; thus, all were mechanosensitive fibers. The conduction velocity of the axon was determined by monopolar or bipolar electrical stimulation through an epoxy-coated electrode with an uninsulated tip. The supramaximal square wave pulses (0.2-2 ms duration; 0.5 Hz) were delivered at the mechanosensitive site of the receptive field. The distance between receptive field and the recording electrode (conduction distance) was measured and divided by the latency of the action potential. The single fibers were classified as being either C- or A-fibers if their conduction velocity was slower than 1.2 m/s or between 1.2 and 8.0 m/s, respectively. The fast conducting (greater than 8.0 m/s) and rapidly adapting fibers were excluded. In this study, C-fibers were investigated for mechanical and heat responses; A-fibers surrounding the incision were included only for mechanical response experiments. In previous experiments [11,13], C-fiber afferents with receptive fields within 2 mm of the incision were sensitized to heat and acid. We emphasized recording fibers from sham control skin and fibers innervating ≤ 2 mm from the incision. Some fibers from incised skin had receptive fields > 2 mm from the incision, these were analyzed separately.

### Recording protocol

#### Ongoing activity

The mechanosensitive receptive field of each fiber was isolated by placing a hollow metal cylinder with silicone grease which was added to prevent leakage from the bath into the receptive field. After a 2 min baseline recording, the SIF solution inside the ring was replaced with C5a (1 or 0.1 μg/ml) or PBS-vehicle in a 100 μl volume and fiber activity was recorded for 2 min.

In other experiments, after a 2 min baseline recording, the SIF solution inside the ring was replaced with PMX53 1.8 μg/ml in a 50 μl volume. After a 2 min exposure to PMX53, C5a 2 μg/ml or PBS-vehicle in a 50 μl volume was added into the ring so that the final concentration of PMX53 was 0.9 μg/ml combined with C5a 1 μg/ml (final concentration) or PBS-vehicle in a total volume of 100 μl. Then fiber activity was recorded for 2 min.

The fiber was considered activated by the drug if activity of at least 0.1 impulse/s was elicited if background activity was absent during baseline recording, or if ongoing activity was present, an increase of at least two standard deviations greater than the average baseline ongoing activity. For PMX53 application, some fibers had ongoing activity. If ongoing activity was present we noted if PMX53 decreased this activity. If the average activity decreased by at least two standard deviations from the baseline, it was considered a significant decrease.

#### Heat responses

After ongoing activity of C-fibers was assessed, the test solution inside the ring was aspirated to minimize heat dissipation and a thermocouple was gently placed to measure the subcutaneous temperature. A radiant heat lamp was placed in the translucent area underneath the organ bath and the light beam was focused onto the epidermal side of the skin. After 10 s baseline recording, a computer-controlled standard heat ramp was delivered starting from 32 to 45°C over 15 s by a customized heat stimulator (Bioengineering, University of Iowa, Iowa City, IA). The peak temperature, 45°C, was used to avoid potential tissue damage of higher temperatures. We previously determined that the temperature of the epidermal side was approximately 1°C higher than the subdermal side. Fibers having a receptive field in a previously heated area were avoided for subsequent recordings. Each fiber was tested with heat only once because previously we noted that repeated heating sensitized the fiber responses. Action potentials were counted for 20 s beginning from the onset of the heat, including the heat ramp application (15 s) and 5 s after the peak temperature when the temperature remained elevated to 41°C, for 'during heat' analysis. Action potentials between 20 s and 60 s after the onset of the heat were also counted for 'after heat' analysis that reflect after discharges following the stimulus. The temperature that elicited the first action potential during heat stimulation was considered as threshold if background activity was absent. If background activity was present, threshold was determined by the temperature during heating that increased background activity at least two standard deviations greater than the average baseline (10 s, 1 s bin). Background activity was subtracted from total action potential numbers during recording period, assuming background activity was sustained during the heat stimulus.

#### Mechanical responses

To avoid repeated application of drug for testing of heat and mechanical stimuli, mechanical responses were tested in separate experiments from heat. Servo-controlled mechanical stimulation (Series 300B dual mode servo system, Aurora Scientific, Canada) was used to measure mechanosensitivity of C- and A-fibers. The most sensitive spot of the receptive field of each fiber was isolated with a metal cylinder as described above. A flat and cylindrical metal probe (tip size 0.7 mm) attached to the tip of stimulator arm was placed onto the receptive field so that no force was generated. After a 2 min baseline recording, a computer-controlled ascending series of square force stimuli (5, 10, 20, 40 and 80 mN; 2 s duration; 60 s intervals) was applied to the receptive field. Then, the SIF solution inside the ring was replaced with C5a 1 μg/ml or PBS-vehicle solution with 100 μl volume without any movement of the mechanical probe or ring. After a 2 min drug application, the same series of mechanical stimuli was applied to the receptive field in the presence of applied drug. C-fibers were tested regardless of location of the receptive fields in relation to the incision; A-fibers ≤ 2 mm from the incision were studied. The force that elicited the first action potential during mechanical stimulation was considered threshold if background activity was absent. If background activity was present, threshold was determined by the force that increased activity by at least two standard deviations greater than the average background. Background activity was subtracted from the total number of action potentials during mechanical stimulation.

### Data analysis

Parametric and non-parametric analyses were selected based on the Kolmogorov-Smirnov test for normal distribution (SigmaStat software. Jandel Corporation, San Rafael, CA). For electrophysiological data, all data were analyzed based on distances between receptive field and incision site: sham *vs. *incision ≤ 2 mm *vs. *incision > 2 mm group based on our previous studies [11,13]. The heat response threshold, and total action potentials during and after heat stimulation were analyzed using t-test (two groups) and one-way analysis of variance (ANOVA) followed by Holm-Sidak correction (three groups) for parametric data analysis, or Mann-Whitney rank sum test (two groups) and Kruskall-Wallis ANOVA followed by Dunn's test (three groups) for non-parametric data analysis. A chi-square test was used to compare the percentage of fibers that had increased ongoing activity or to evaluate the proportion of afferents responding to heat and the proportion that changed background activity. Analysis of the mechanical stimulus-response relationship before and after drug was performed using two-way repeated measures ANOVA. Electrophysiological data for heat and mechanical data are presented as median value and means ± SEM, respectively. The behavioral and mRNA expression data were analyzed by two-way ANOVA followed by Bonferroni post-hoc test for multiple comparisons. Protein analysis was done by one-way ANOVA followed by Bonferroni post-hoc test for multiple comparisons. Behavior data and the expression of mRNA and protein are presented as means ± SEM. *P *values less than 0.05 were considered significant (Prism 4, GraphPad Software, La Jolla, CA).

## Results

### Behavior

#### PMX53 suppresses heat hyperalgesia and mechanical allodynia induced by hind paw incision

**Figure 1:**
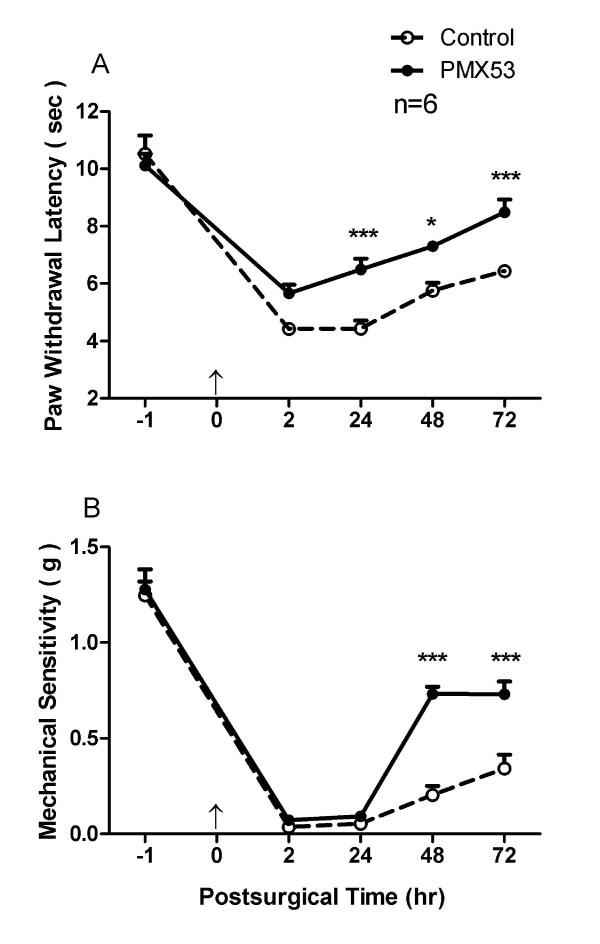
**The effects of C5aR antagonist PMX53 on heat and mechanical nociception after incision**. Intraplantar injection of PMX53 (30 μg/15 μl) reduced heat hyperalgesia and mechanical allodynia induced by hind paw incision (A and B, respectively). The first injection of PMX53 was administered before paw incision, the second and subsequent administrations were made 2 hours prior to each test session. For panels A and B values are expressed as mean ± SEM, *, *P *< 0.05 or ***, *P *< 0.001 compared to the control group. An arrow head at time = 0 indicates incision.

Heat hyperalgesia and mechanical allodynia are important features of tissue injury related to surgery and trauma [26,27]. Paw incision induced significant heat hyperalgesia during the 72 hours following incision as displayed in Figure 1A. However, local administrations of the C5aR antagonist PMX53 via intraplantar injection before and after incision significantly attenuated this sensitization in comparison with the control group. Likewise in our experiments, mechanical allodynia was evident for the entire 72 hour period over which incised mice were observed as shown as Figure 1B. Intraplantar injection of PMX53 reduced the mechanical allodynia from 48 to 72 hours after incision, but not at earlier time points post incision.

#### PMX53 suppresses heat hyperalgesia and mechanical allodynia induced by C5a

**Figure 2:**
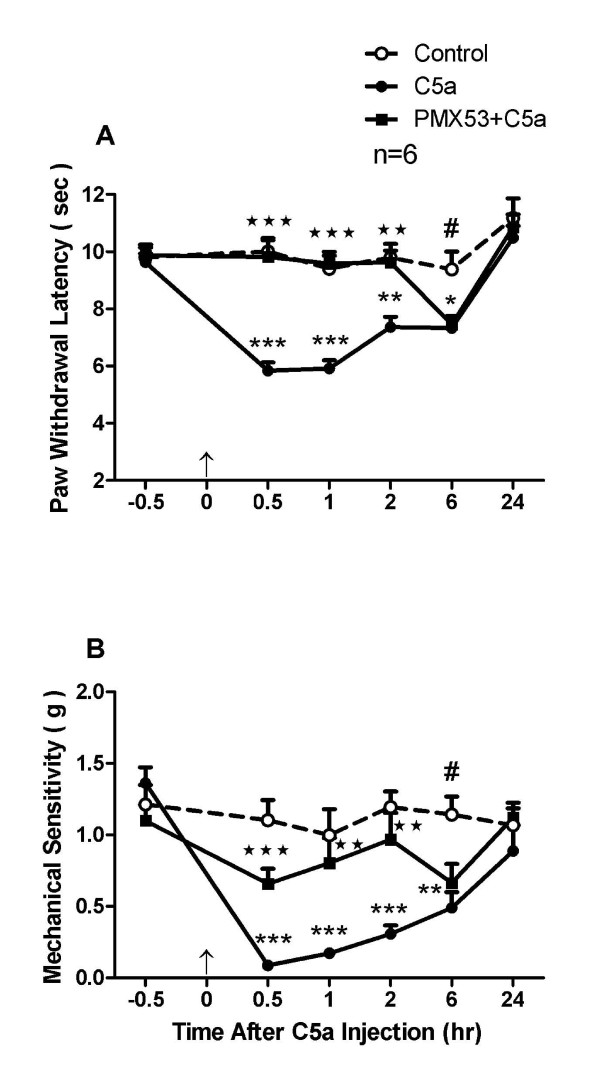
**Pre-treatment with PMX53 (3 mg/kg) abolished the heat hyperalgesia (A) and mechanical allodynia (B) induced by intraplantar injection of recombinant mouse C5a (200 ng)**. Values are expressed as mean ± SEM, *, *P *< 0.05, **, *P *< 0.01 or ***, *P *< 0.001 (comparison of C5a group and saline group); #, *P *< 0.05 (comparison of PMX53 group and saline group); ⋆⋆, *P *< 0.01 and ⋆⋆⋆, *P *< 0.001 (comparison of PMX53 and C5a groups). An arrow head at time = 0 indicates C5aR agonist injection.

The results from the local injection of PMX53 suggest that C5a present in skin can cause heat and mechanical nociceptive sensitization. We attempted to confirm this using local C5a injection. Intraplantar injection of recombinant C5a induced significant heat hyperalgesia lasting at least 6 hours as displayed in Figure 2A. The peak level of sensitization was within the first 1 hour, with the slow return to baseline at 24 hours after C5a injection. However, pre-treatment with the PMX53 (3 mg/kg) largely abolished this heat sensitivity for the first 2 hours after C5aR agonist injection. Intraplantar injection of C5a significantly induced mechanical allodynia along a time course similar to the heat effects as displayed in Figure 2B. The peak effect was reached within 0.5 hour after C5a injection, then slowly returned to the baseline level at 24 hours. However, pre-treatment with C5aR antagonist again prevented this sensitization for about 2 hours after C5a injection.

### C5a and C5aR mRNA

**Figure 3:**
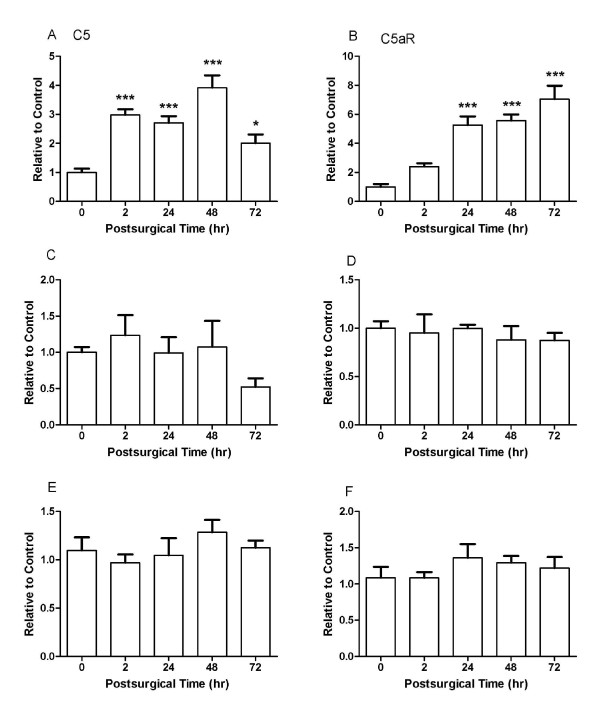
**Incision alters levels of C5 and C5aR after hind paw incision**. Samples for C5 and C5aR mRNA analyses were collected at the indicated time points. Data are displayed for peri-incisional skin (A and B), spinal cord (C and D) and DRG (E and F). For each tissue n = 4 samples, each analyzed in triplicate. Statistical comparisons were made between normalized baseline expression levels and those measured at the indicated time points. Data are presented as mean ± SEM. *, *P *< 0.05, ***, *P *< 0.001.

Peri-incisional skin was analyzed for C5a and C5aR expression (Figure 3A-F). The C5 mRNA in skin was significantly increased as early as 2 h and reached the peak at 48 h after incision. Also C5aR mRNA in skin gradually increased after incision and peaked with a 7-fold increase at 72 h. There were no significant changes of C5 or C5aR mRNA in DRG or spinal cord samples after incision.

### C5a production in Skin

**Figure 4:**
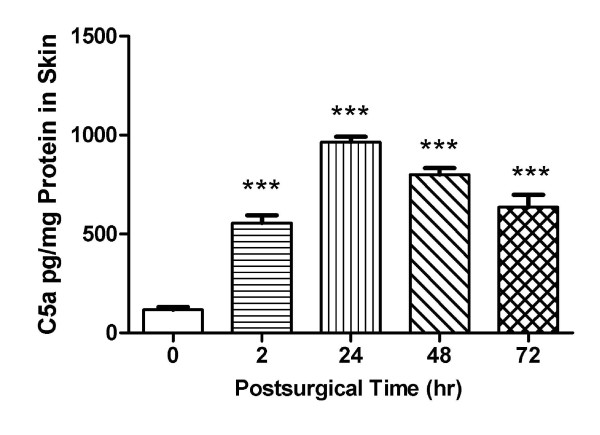
**C5a level in wounded skin was analyzed by a murine C5a ELISA**. C5a level was significantly increased at the indicated time points after incision in comparison with control group (time 0). Values are displayed as the mean ± SEM, n = 5, ***, *P *< 0.001.

In the previous experiment we documented that C5a precursor, C5 mRNA level was increased in the incised skin. To further define the role of C5a in postsurgical pain we established a mouse C5a ELISA procedure to directly detect C5a production in the skin tissue. The data in Figure 4 demonstrate that C5a production increased in peri-incisional skin from 2 h at least 72 h after the incision in comparison with control non-incision group (*P *< 0.001).

### Single fiber recording

#### Ongoing activity

**Figure 5:**
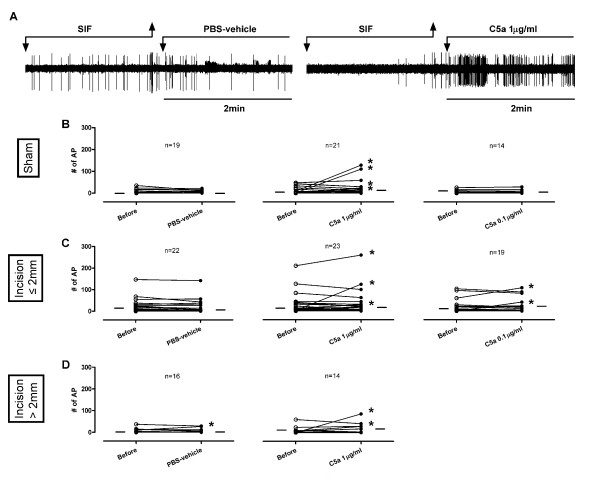
**Effects of C5a on ongoing activities of mechanosensitive C-fibers in incised tissue**. (A) Example recordings of C-fibers with receptive fields ≤ 2 mm from the incision before and after application of PBS-vehicle or C5a 1 μg/ml. Artifacts are produced during replacement of solutions as marked. (B-D) Ongoing activities of C-fibers before and after exposure to C5a (1 or 0.1 μg/ml) or PBS-vehicle which have receptive fields ≤ 2 mm (C) and > 2 mm (D) from the incision and in the sham group (B). Each line on the graphs represents a single unit. * indicates an increase of ongoing activity after drug application. The small horizontal lines in each graph indicate median values.

Application of PBS-vehicle did not increase ongoing activity in C-fibers in the sham group (0/19) or in afferents from the incised tissue with receptive fields ≤ 2 mm from the incision (0/22) (Figure 5A-D). C5a 1 μg/ml increased ongoing activity in 4 of 21 (19.0%) C-fibers in the sham group. Afferents from incised tissue were similarly influenced; 3 of 23 (13.0%) C-fibers ≤ 2 mm from the incision and 2 of 14 (14.2%) C-fibers > 2 mm from the incision had increased activity. Similar to previous studies [6], C5a 0.1 μg/ml did not excite fibers in the sham group (0/14); only 2 of 19 (10.5%) C-fibers ≤ 2 mm from the incision increased activity. A significant difference (*P *= 0.034) was only observed in the proportion of C-fibers activated by C5a 1 μg/ml (4/21, 19.0%), C5a 0.1 μg/ml (0/14) and PBS-vehicle (0/19) in the sham group; however, post hoc tests did not reveal a significant difference of C5a *vs. *PBS-vehicle. No significant difference was observed in the proportion of C-fibers activated by C5a 1 μg/ml (3/23, 13.0%), C5a 0.1 μg/ml (2/19, 10.5%) and PBS-vehicle (0/22) that had receptive fields ≤ 2 mm from the incision. Thus, there was no evidence that more C-fibers were activated by C5a in incised skin versus the sham preparations.

In this preparation, 14 of 38 (36.8%) fibers treated with PBS-vehicle had significant spontaneous activity (at least 0.1 impulse/s); activity decreased in 2 of 14 spontaneously active fibers (14.2%) after PBS-vehicle. Of those exposed to PMX53 1.8 μg/ml, 9 of 42 (21.4%) fibers were spontaneously active; only 3 of 9 spontaneously active fibers (33.3%) decreased ongoing activity after PMX53 application (data not shown). For all spontaneously active C-fibers, regardless of proximity to the incision, no differences were observed in the proportion that decreased activity after PMX53 *vs. *PBS-vehicle. Thus, there was no apparent contribution of C5a to this ongoing activity that is present *in vitro *based on responses to PMX53.

**Figure 6:**
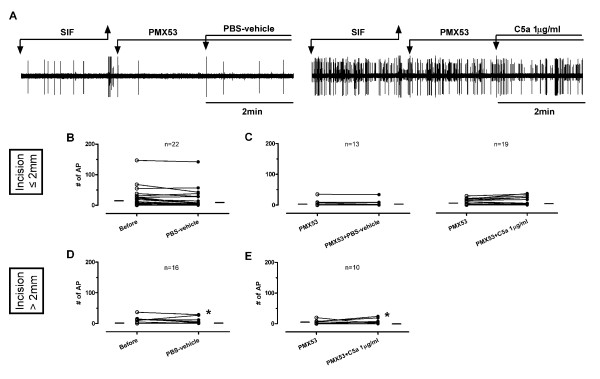
**Effects of PBS-vehicle and C5a applied after PMX53 on ongoing activities of mechanosensitive C-fibers in incised tissue**. (A) Example recordings of C-fibers with receptive fields ≤ 2 mm from the incision before and after application of PMX53 plus PBS-vehicle or PMX53 plus C5a 1 μg/ml. After recording of baseline, PMX53 1.8 μg/ml was applied, then PBS-vehicle or C5a was added to make the final concentration of PMX53 0.9 μg/ml. Artifacts are produced during replacement of solutions as marked. (C and E) Ongoing activities of C-fibers before and after exposure to PMX53 0.9 μg/ml plus PBS-vehicle or PMX53 0.9 μg/ml plus C5a 1 μg/ml which have receptive fields ≤ 2 mm (C) and > 2 mm (E) from the incision. For comparison, PBS-vehicle data from Figure 5C and D are in used in Figure 6B and D. Each line on the graphs represents a single unit. * indicates an increase in ongoing activity by drug application. Small horizontal lines in each graph indicate median values.

When C5a 1 μg/ml was applied after PMX53, 0 of 19 fibers with receptive field ≤ 2 mm from the incision and only one fiber with receptive field > 2 mm from the incision (1/10, 10.0%) increased ongoing activity (Figure 6A-E). PMX53 pre-treatment blocked C5a-induced excitation (Figure 6). However, because few fibers were directly excited by C5a (Figure 5); the difference in proportion of fibers activated by C5a between the two groups (control *vs*. PMX53) did not reach the statistical significance (Figure 5*vs. *Figure 6).

#### Heat responses

**Figure 7:**
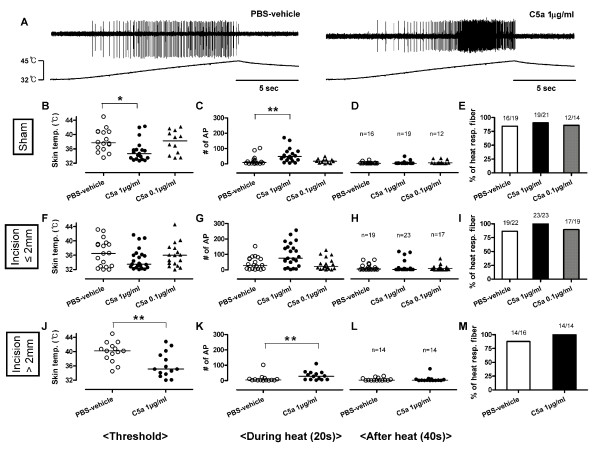
**Effects of C5a on heat responses of mechanosensitive C-fibers in incised tissue**. (A) Example recordings of C-fibers with receptive fields ≤ 2 mm from the incision after application of PBS-vehicle or C5a 1 μg/ml. (B-D, F-H and J-L) Heat responses of C-fibers after exposure to C5a (1 or 0.1 μg/ml) or PBS-vehicle which have receptive fields ≤ 2 mm (F-H) and > 2 mm (J-L) from the incision and in the sham group (B-D). The proportion of C-fibers responsive to heat after application of each drug (E, I and M). Heat thresholds (B, F and J) and total action potentials for 20 s during heat application (C, G and K) and 40 s after heat application (D, H and L). Each symbol represents a single unit. *, *P *< 0.05. **, *P *< 0.01 *vs *PBS-vehicle. Data are presented as median values.

In the sham group, C5a 1 μg/ml sensitized C-fibers to heat (Figure 7A-M) by lowering the heat threshold and increasing the number of action potentials during heat stimulation compared to PBS-vehicle (Figure 7B and 7C). C5a 0.1 μg/ml had no effect. In C-fibers with receptive fields ≤ 2 mm from the incision, C5a 1 μg/ml and 0.1 μg/ml did not significantly sensitize responses to heat versus PBS-vehicle (Figure 7F and 7G). In C-fibers with receptive fields > 2 mm from the incision, C5a 1 μg/ml sensitized C-fibers to heat by lowering the heat threshold and increasing the number of action potentials during heat stimulation (Figure 7J and 7K) as in the sham tissue. The after heat responses were not remarkable in any group (Figure 7D, H and 7L).

In the PBS-vehicle treated tissue, a high percentage of C-fibers (49/57, 85.9%) in all groups responded to heat (Figure 7E, I and 7M). There was no difference in the proportion of fibers responding to heat among the sham group and those with receptive fields ≤ 2 mm and > 2 mm from the incision. Almost all C-fibers (85/91, 93.4%) responded to heat after application of C5a (1 or 0.1 μg/ml), however, incision, compared to sham, did not increase the heat responses to C5a or the proportion of C-fibers responding to heat.

**Figure 8:**
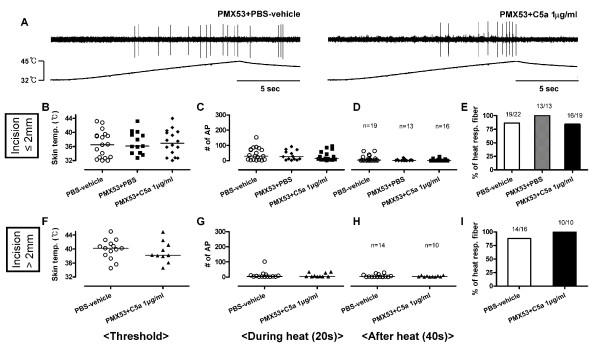
**Effects of PMX53 pre-treatment on heat responses of mechanosensitive C-fibers in incised tissue**. (A) Example recordings of C-fibers with receptive fields ≤ 2 mm from the incision after application of PMX53 0.9 μg/ml plus PBS-vehicle or PMX53 0.9 μg/ml plus C5a 1 μg/ml. (B-D and F-H) Heat responses of C-fibers after exposure to PMX53 0.9 μg/ml plus PBS-vehicle or PMX53 0.9 μg/ml plus C5a 1 μg/ml which have receptive fields ≤ 2 mm (B-D) and > 2 mm (F-H) from the incision. The proportion of C-fibers responsive to heat after application of each drug (E and I). Heat thresholds (B and F) and total action potentials for 20 s during heat application (C and G) and 40 s after heat application (D and H). PBS-vehicle data B-I are from Figure 7F-M. Each symbol represents a single unit. Data are presented as median values.

In C-fibers with receptive fields ≤ 2 mm from the incision (Figure 8A-E), the combination of PMX53 and PBS-vehicle did not affect the heat responses of C-fibers compared to PBS-vehicle alone (vehicle data from Figure 7F-I). When C5a 1 μg/ml was applied after PMX53, the heat threshold, total number of action potentials during and after heat stimulation, and the proportion of heat responsive C-fibers were not different compared to PBS-vehicle. Similarly, in the C-fibers with receptive fields > 2 mm from the incision (Figure 8F-I), C5a did not affect heat responses (*vs. *PBS-vehicle, data from Figure 7J-M) when the receptive fields were treated with PMX53. Thus, PMX53 by itself did not affect heat responses of C-fibers with receptive fields ≤ 2 mm from the incision, but inhibited heat sensitization induced by C5a in C-fibers with receptive fields > 2 mm from the incision.

#### Mechanical response

**Figure 9:**
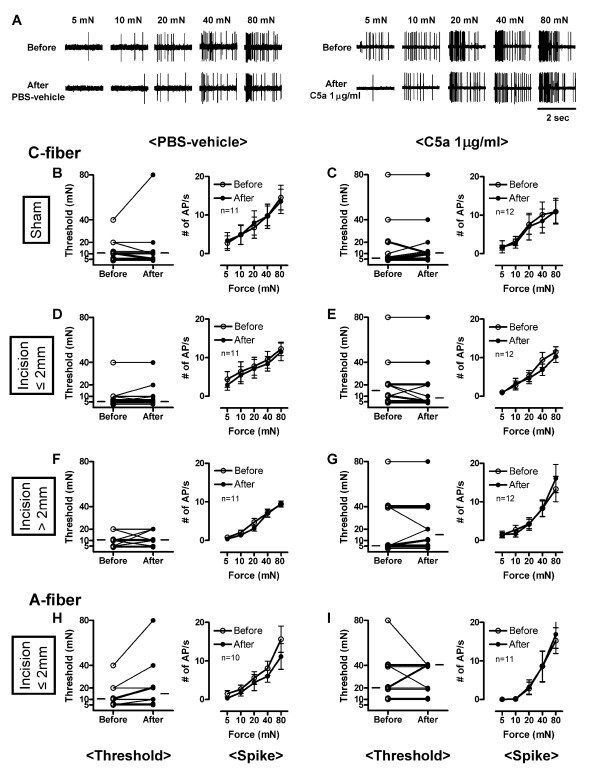
**Effects of C5a on mechanical responses of mechanosensitive C- and A-fibers in incised tissue**. (A) Example recordings of C-fibers with receptive fields ≤ 2 mm from the incision before and after application of PBS-vehicle or C5a 1 μg/ml. (B-G) Mechanical thresholds (left panels) and stimulus response function (right panels) of C-fibers before and after exposure to PBS-vehicle or C5a 1 μg/ml which have receptive fields ≤ 2 mm (D and E) and > 2 mm (F and G) from the incision and in sham group (B and C). (H and I) Mechanical thresholds (left panels) and stimulus response function (right panels) of A-fibers before and after exposure to PBS-vehicle or C5a 1 μg/ml which have receptive fields ≤ 2 mm from the incision. In left panels, each line represents a single unit, and the small horizontal lines in each graph indicate median values. In right panels, data are presented as means ± SEM.

The mechanical thresholds were not decreased by C5a 1 μg/ml or PBS-vehicle in C- or A-fibers from both sham and the incision groups (Figure 9A-I). The median mechanical thresholds of C-fibers before *vs. *after exposure to C5a 1 μg/ml were as follows: sham (5 *vs. *10 mN), incision ≤ 2 mm (15 *vs. *7.5 mN) and incision > 2 mm (5 *vs. *15 mN). Similarly, neither PBS-vehicle nor C5a 1 μg/ml affected mechanical threshold of A-fibers with receptive fields ≤ 2 mm from the incision. The median values of threshold of A-fibers before *vs. *after exposure to C5a 1 μg/ml, which had receptive fields ≤ 2 mm from the incision, were 20 *vs. *40 mN. The stimulus response functions were not enhanced by C5a 1 μg/ml or PBS-vehicle. Incremental mechanical forces (5, 10, 20, 40 and 80 mN) applied on the receptive field elicited greater responses. The activity during mechanical stimulation was not influenced by PBS-vehicle or C5a 1 μg/ml in C-fibers from sham or incised skin. Similarly, neither PBS-vehicle nor C5a 1 μg/ml enhanced responses to mechanical stimuli of A-fibers with receptive fields ≤ 2 mm from the incision. Thus, the responses to mechanical stimuli were not sensitized by C5a *vs. *PBS-vehicle in C- or A-fibers from sham and incised tissue.

## Discussion

In this study, mRNA levels of C5 and C5aR, and C5a protein in the skin were dramatically increased after incision. Blockade of C5aR *in vivo *using a selective C5aR antagonist, PMX53, decreased exogenous C5a-induced heat and mechanical hypersensitivity. PMX53 alleviated incision-induced heat hypersensitivity at 24 hours and decreased mechanical hypersensitivity later at 48 hours. *In vitro *PMX53 blocked sensitization of C-fiber afferents to heat by C5a but did not by itself influence ongoing activity or heat sensitivity in afferents innervating control or incised skin. C5a sensitized C-fiber afferent responses to heat; this was less evident in afferents adjacent to the incision. C5a did not elicit a greater prevalence of increased ongoing activity in afferents from incised versus unincised control tissue. The magnitude of mechanical responses was also not affected by C5a in any nociceptive fibers innervating incised or unincised skin. Altogether, high local concentrations of C5a contribute to enhanced behavioral responses to heat 1 day after incision and also to mechanical responses on day 2 and thereafter. High local C5a expression rather than increased responsiveness of nociceptors to C5a contributes to hypersensitivity after incision.

### Behavior responses

The hind paw incision induced heat and mechanical hypersensitivity, and these hypersensitivities were significantly attenuated by local blockade of C5aR in the hind paw (Figure 1), highlighting the contribution of peripheral C5a to incisional pain. Heat, but not mechanical sensitization was reduced by C5aR blockade early after incision, i.e. within the first 24 hours. PMX53 was also able to suppress behavioral hyperalgesia induced by local injection of C5a (Figure 2). Hyperalgesia caused by the application of other inflammatory mediators, e.g. zymosan, carrageenan and lipopolysaccharide have also been associated with C5aR activation [37]. Our studies here suggest that at least at early time points, C5a alters the heat sensitivity of C-fibers more markedly than mechanical sensitivity. Apparently the contribution of peripheral C5a signaling to mechanical sensitization is a relatively minor contributor to sensitization early as opposed to later times after incision. The contribution of central C5a in neuropathic pain has been suggested [28,29]; however it remains to be determined whether it also holds true for postoperative pain.

### mRNA and protein expression

The mRNA levels of C5 and C5aR after incision were locally plastic, but we did not find evidence for C5 system plasticity within the DRG or central nervous systems (Figure 3). While C5 levels near incisions peak early and remain elevated (Figure 3 and 4), local C5aR changes only become significant at about 24 hours after incision. Though we have shown C5a can act directly on afferent fibers [6], we observed no increase in C5aR expression in the DRG or spinal cord after incision. Thus, the up-regulation of C5 and C5aR after incision appears to be locally restricted, and perhaps related to the known expression of C5/C5aR by neutrophils and intrinsic cells of the skin.

### Electrophysiology

Application of C5a to nociceptive terminals directly activated few C-fibers in both sham and incised tissue (Figure 5); therefore enhanced activation by C5a on incision is not evident. Based on the lack of effect of PMX53 applied to the receptive field of spontaneously active fibers (Figure 6), no significant contribution of C5a to the ongoing activity in the incised skin nerve preparation was observed.

In previous studies, incision sensitized nociceptors to heat and lactic acid in skin [11,30]. Afferents in muscle were also sensitized by incision; heat, mechanical and acid responsiveness were enhanced compared to unincised muscle [31]. It was surprising that incision did not enhance the ability of C5a to sensitize C-fiber afferents to heat or produced spontaneous activity. In fact responses to C5a were not significantly different than vehicle in afferents ≤ 2 mm from the incision. The lack of significance may be due to the fact that some fibers were already sensitized to heat by incision [30], and the effects of additionally applied C5a on heat responses on afferents adjacent to the incision may be saturated. Because the heat responses (e.g. threshold and total number of action potentials during and after heat) tend to be sensitized in afferents adjacent to the incision (Figure 7F-H), the study may be underpowered to detect a further significant change by C5a. It is possible that mechanically insensitive fibers contribute to heat hyperalgesia in incised and control tissue as well. Some mechanically insensitive fibers could become mechanosensitive after incision, and contribute to heat responses; however, this can not be determined in this protocol. Based on the heat responses after PMX53 application alone (Figure 8), the C5aR does not contribute to heat responses of C-fibers in the incised *in vitro *preparation.

C5a-induced mechanical hyperalgesia occurs *in vivo *in naïve animals (Figure 2B). In the present study and in a previous study, neither C-fibers (Figure 9C) nor A-fibers sensitized to mechanical stimuli in sham control tissue [6], and C5a did not affect mechanical responsiveness of C- or A-fibers in skin from mice that had undergone incision 24 h earlier (Figure 9). Mechanical hypersensitivity may be secondary to other *in vivo *elements activated by complement which are removed in the skin nerve preparation. Also, the brief treatment period *in vitro *may not be sufficient to cause mechanical sensitization that occurs as early as 30 min after complement injection *in vivo*. Finally, because mechanical sensitization of nociceptors has been less apparent than heat sensitization in several studies, many propose that mechanical hypersensitivity is in part due to sensitization in the central nervous system [32].

### The possible role of C5a in incisional pain

Based on observations that C5aR mRNA is expressed in primary sensory neurons and the intracellular calcium level is elevated by C5a treatment in cultured DRG neurons *in vitro *[6], behavioral sensitization after incision could be caused by a direct binding of C5a to its peripheral receptors. At the same time, it is also possible that C5a plays a role in incisional pain by indirect mechanisms. C5a is one of the most potent inflammatory molecules known to induce proinflammatory functions such as upregulation of cytokine and chemokine production, and it is also a strong chemoattractant for neutrophils, monocytes and macrophages that can release a mixture of algogenic substances, e.g. histamine, interleukins, TNF-α and others [1-4,33,34] which produce heat and mechanical hyperalgesia when injected into hind paw [35-41]. In support, mRNA levels of peripheral C5 and C5aR were markedly increased in the incised skin but not DRG or spinal cord after incision (Figure 3), implying the recruitment of inflammatory cells to the incision site. Our previous study showed several locally produced cytokines from incisions were dependent on C5aR [10]. Thus, these indirect effects of C5a on nociceptive sensitization may be as important as the effects of C5a itself on sensory neurons.

Additionally, activation of small diameter nociceptors by C5a may contribute to behavioral sensitization by releasing various algesic substances e.g. glutamate, neuropeptides and ATP in the peripheral and central terminals to sensitize dorsal horn neurons [42-45] and nociceptors [46-50]. However, this does not occur immediately after application to skin *in vitro*. It is possible that application of C5a for several hours *in vitro *could induce mechanical sensitization; however, this can not be tested.

## Conclusions

In summary, treatment with a selective C5aR antagonist, PMX-53, decreased heat and mechanical hypersensitivity induced by incision or by local administration of C5a *in vivo*. The increase of C5a protein level in the incised skin was observed. The mRNA levels of C5 and C5aR in the skin were increased after incision, but not in the DRG and spinal cord. The sensitivity of nociceptors to C5a was not significantly enhanced by incision *in vitro*. Therefore, the reduced hypersensitivity resulting from PMX53 administration *in vivo *is caused by blockade of receptor activation produced by high local concentrations of C5a. In the future, the effect of drugs like PMX53 on clinical postoperative pain could be considered.

## Competing interests

The authors declare that they have no competing interests.

## Authors' contributions

This study is based on the original idea of JDC and TJB. JHJ carried out the electrophysiology studies and drafted the manuscript. DL carried out behavioral studies and molecular biology. KK participated in electrophysiology. YS performed mRNA studies. JHJ and DL performed data analyses. TJB and JDC were responsible for supervising entire experiments, data analyses and writing manuscript. All authors read and approved the final manuscript.
